# Association between a syndemic of psychosocial problems and unprotected anal intercourse among men who have sex with men in Shanghai, China

**DOI:** 10.1186/s12879-016-2132-8

**Published:** 2017-01-07

**Authors:** Ying Wang, Zezhou Wang, Mengmeng Jia, Ajuan Liang, Dong Yuan, Zhe Sun, Feng Gan, Yichen Wang, Yong Cai, Zhiruo Zhang

**Affiliations:** 1 School of Public Health, affiliated with School of Medicine, Shanghai Jiao Tong University, Shanghai, 200025 China; 2 Renji Hospital, affiliated with School of Medicine Shanghai Jiao Tong University, Shanghai, 200127 China; 3 Shanghai Municipal Center for Disease Control and Prevention, Shanghai, 200336 China; 4 School of Continuing Education, Shanghai Jiao Tong University, Shanghai, 200030 China; 5 Ruijin Hospital, affiliated with School of Medicine, Shanghai Jiao Tong University, Shanghai, 200032 China

**Keywords:** MSM, Psychosocial problems, Syndemic

## Abstract

**Background:**

Previous studies have discussed the overlapping and reinforcing effects (defined as a syndemic) of psychosocial problems on high-risk sexual behaviors among men who have sex with men (MSM). The present study aimed to apply the syndemic theory to verify the reinforcing effects of psychosocial problems on unprotected anal intercourse (UAI) among MSM in Shanghai, and determine if other important psychosocial factors fit into the syndemic theory.

**Methods:**

Data were collected from 547 MSM in Shanghai, China, through face-to-face interviews. The measures for psychosocial problems included the Rosenberg Self-Esteem Scale; the Generalized Anxiety Disorder-7; the Center for Epidemiological Studies Depression Scale; the University of California, Los Angeles Loneliness Scale; and the Sexual Compulsivity Scale. We used multivariate analysis and binary logistic regression to investigate the associations between psychosocial problems and high-risk sexual behaviors.

**Results:**

The prevalence of UAI among MSM in the past 6 months was 54.5%. Education (graduate from college vs. high school) served as a protective factor against UAI (OR 0.59, 95% CI: 0.38–0.94). There was a high prevalence of psychosocial problems, and at least one-third of participants reported experiencing more than two psychosocial symptoms. Of these psychosocial factors that we investigated, lower self-esteem was associated with UAI in both univariate and multivariate regression model (*P* = 0.009). Result suggests that overlapping and reinforcing effects of psychosocial problems may increase high risk sexual behaviors among MSM in Shanghai, China (OR 1.65, 95% CI: 1.09–2.50; *P* = 0.018).

**Conclusions:**

We found further evidence for a syndemic of psychosocial problems among MSM in Shanghai, China. This syndemic may also increase high risk sexual behaviors among MSM. Most HIV prevention interventions are focused on behavior change and only have moderate effects; our findings suggest that a shift from behavior-focused interventions to a more comprehensive strategy that addresses psychosocial factors may be necessary.

## Background

The group of men who have sex with men (MSM) accounts for the largest number of new HIV infections [1]. The HIV infection rate in the Asian MSM population has dramatically increased in recent years, and unprotected anal intercourse (UAI) and multiple sexual partners (MSP) are considered to be the leading risk factors for HIV infection among MSM [2–5]. This is also the case in China, and as MSM who have UAI with men may also have unprotected sex with women, there is a risk of spreading HIV to the general population [6]. There are an estimated 10 to 25 million MSM in China; estimates of the HIV prevalence due to high risk sexual behaviors (especially UAI) in the MSM population range from 6.5 to 7.2% [6–8]. When it comes to high-risk sexual behaviors among MSM, it mainly means UAI and MSP. Of those living with HIV/AIDS in China, an estimated 27.2% were infected via unprotected sexual intercourse among MSM [9, 10].

The MSM population may not be accepted by most Chinese families. The pressure of being a minority group discriminated by mainstream society may cause psychosocial problems, such as depression and anxiety [1, 2, 11, 12]. Estimates of the prevalence of depression in the MSM population ranged from 29.2 to 63.9%, which were mostly due to social acceptance for homosexuality and other factors such as HIV-related stigma, whereas in the general population the prevalence was 5.3 to 23% [13]. Other psychosocial problems such as anxiety have also been suggested to be more prevalent among MSM than in the general population [14].

Some studies have noted that the high-risk sexual behaviors of MSM such as UAI were highly correlated with their psychosocial problems, and these psychosocial problems might contribute to HIV vulnerability [9, 15, 16]. Serious psychosocial problems may also lead to high-risk sexual behaviors. Research on MSM in India showed that MSM with depressive symptoms tended to participate in UAI [17]. Results from the article written by De Santis JP and Wilson PA indicated that higher levels of depressive symptoms had a statistically significant relationship to higher levels of high-risk sexual behaviors such as UAI. They suggested that increases in depression among sexually active MSM may result in externalizing symptoms such as sensation-seeking and the use of sex to avoid or alleviate negative affect [18, 19]. Similar conclusions were drawn in studies with MSM in Spain, rural areas, and in older adults [18, 20, 21]. Psychosocial problems may also be obstacles that prevent the MSM population from using health services. Research indicates that MSM with psychosocial problems usually do not visit a doctor even if when they are unwell [6, 22]. Similarly, researchers in China concluded that psychosocial problems may increase the vulnerability to HIV among MSM [9, 23]. It is important to understand the effect of co-occurring psychosocial problems on behavior as well. Psychosocial problems may interact with each other (syndemic) and increase the risk of HIV infection [2, 17, 24–26]. A syndemic is defined as synergistically interacting health problems coming together and producing additional disease in a population; the various factors may have an additive effect and intensify each other [16, 27]. This was first found by Stall when he surveyed MSM in four major American cities [26]. And the United States Center for Disease Control and Prevention (CDC) defined syndemic as more than two factors interacting with each other, and potentially causing an extra burden on people [28, 29]. A number of studies have shown that psychosocial problems were syndemic factors which might lead to high-risk sexual behaviors among MSM [6, 30, 31]. High-risk sexual behaviors have also been found to be highly correlated with the number of psychosocial problems [15–17, 32]. Another survey found that the more frequently MSM reported participating in UAI, the more psychosocial problems there were [33].

Most research on MSM in China has focused on demographic characteristics, sexual behaviors, and HIV infection [34, 35]; only a small number of studies have explored the psychosocial context [36, 37], and even fewer have considered the interacting and overlapping effects of psychosocial factors [11, 12]. In the present study, we investigated the prevalence of psychosocial problems in MSM in Shanghai, to determine if a syndemic of psychosocial problems could increase high-risk sexual behaviors and HIV infection in this population. Previous studies on the syndemic theory outline a framework consisting of several essential factors: drug use, depression, childhood abuse, and intimate partner violence; all of which were verified in most studies [1, 17, 21, 26, 38]. Other studies have shown that factors such as smoking, heavy alcohol use, anxiety, sexual compulsivity, and suicidal ideation may also fit into the syndemic theory [12, 15, 23, 39]. Self-esteem and loneliness may also be important psychosocial factors for MSM, yet studies about these factors were disproportionately rare [21, 25, 40, 41]. Self-esteem is an important predictor of depressive symptoms, and people with lower self-esteem may experience higher stigma and be more depressed [42]. An association between homophobic stigma and risk-taking behaviors has been found [43]. Another study showed that as loneliness increased, condom use among MSM decreased [44]. Therefore, we chose self-esteem and loneliness as psychosocial factors in addition to anxiety, depression, and sexual compulsivity. We hypothesized that these five co-occurring factors (self-esteem, anxiety, depression, loneliness, and sexual compulsivity) may interact synergistically, reinforce each other, and finally impose an extra burden on MSM in Shanghai, China. Our findings may provide evidence in China for the syndemic theory, and show that different psychosocial factors may interact synergistically to increase HIV risk in the MSM population. If this is the case, attention should be directed to designing tailored, preventive strategies and holistic public health policies targeted to this population subgroup.

## Methods

### Study setting

Shanghai is the economic center of China, and with its fast-growing economy and tolerance for subculture, it attracts MSM from all over the country. Our research group has a long-term cooperative relationship with the Shanghai Center for Disease Control and Prevention, the Shanghai Dermatology Hospital, and the Shanghai Youth AIDS Health Promotion Centre (a non-governmental organization). This cooperative relationship enabled us to conduct our study with MSM in Shanghai. Our target population was men who had sex with men in the past 6 months, regardless of how they identified themselves (e.g., bisexual, homosexual, or heterosexual).

### Study and sampling procedure

The inclusion criteria were men who had sex with men in the past 6 months and were aged over 16 years. As they experience discrimination from mainstream society, most MSM tend to hide their sexual orientation. Therefore, it would be difficult to identify MSM and use a random trial method to conduct our survey. Instead, we used a snow-balling method to recruit eligible participants, and this proved to bean efficient way to approach a minority population. First, with help from the Shanghai Youth AIDS Health Promotion Centre, we located some individuals who met the inclusion criteria and invited them to complete the questionnaire. We then asked these initial participants to introduce other men eligible to take part in the survey. We repeated this procedure until we obtained the necessary sample size.

A review of the literature highlighted that the two most important risk indicators for HIV infection among MSM were UAI and MSP. We selected UAI as the observed variable. The reported prevalence of UAI among MSM varied from 41 to 78% (average of 50%) [9, 32, 38]. Assuming a UAI prevalence of 50%, an α of 0.05 and a relative sampling error of 0.1P, we calculated the necessary sample size as 400. We increased this by 50% to compensate for any sampling error. Given a target response rate of 95%, the final required sample size was 463.

In total, 567 MSM agreed to participate and 547 participants completed the questionnaire (response rate of 96%). Data were collected in face-to-face interviews. Each interview lasted about 30 min, and participants were paid 100 CNY (16.34 USD) before the interview as compensation for travel expenses.

### Statistical analysis

Data were double-entered using Epidata 3.0. Data were analyzed with the Statistical Program for Social Sciences version 20.0 (IBM SPSS Statistics for Windows, Version 20.0. Armonk, NY: IBM Corp.). Descriptive statistics, such as means, standard deviations (SD), frequencies, and percentages were used to examine the socio-demographic characteristics of MSM in Shanghai.

We analyzed data in five steps. First, we described the demographic characteristics and evaluated their association with UAI by univariate logistic regression. Second, we analyzed psychosocial factors and tested their association with UAI by univariate logistic regression after adjusting for statistically significant demographic variables. Third, we used a forward stepwise multivariate logistic regression to evaluate risk psychosocial factors after adjusting for significant demographic variables. Fourth, we conducted binary logistic regression to test the syndemic effects between all psychosocial factors and UAI after adjusting for significant demographic variables. Finally, we also tested the syndemic effects after excluding significant psychosocial factors which were verified in step three. All the significance level was set at 0.05.

### Ethical considerations

We obtained approval for the present study from the Ethics Committee of the School of Public Health Shanghai Jiao Tong University. Background information about the survey was given to all participants, after which they were given written informed consent forms, which set out the goal and procedure of the study, as well as the potential risks. Written informed consent was obtained from all participants before the study began. During the recruitment and interview procedure, participants were free to ask any questions and to withdraw if they did not wish to continue.

### Measures

#### Background characteristics

Socio-demographic characteristics including age, educational level, marital status, monthly income, residential status, self-report sexual orientation, and self-reported HIV status were collected.

#### Psychosocial problems

Psychosocial problems including self-esteem, anxiety disorder, depression, loneliness, and sexual compulsivity were measured using standard scales.

#### Rosenberg self-esteem scale

Self-esteem is correlated with many other indicators of psychosocial health problems, and is considered to core measurement of psychosocial health problems [45]. The Rosenberg Self-Esteem Scale is a reliable and valid tool to assess self-esteem and is widely used in scientific research [46]. It consists of 10 Likert-type items, for example, “I feel that I am a person of worth, at least I am equal to others” [47]. Responses to all items are on a 4-point scale, ranging from strongly agree to strongly disagree. For items 1, 2, 4, 6, and 7, “strongly agree” is scored 3, “agree” is scored 2, “disagree” is scored 1, and “strongly disagree” is scored 0. Items 3, 5, 8, 9, and 10 are reversed (“strongly agree” is scored 0 and “strongly disagree” is scored 3). The score range was 0 to 30 (Cronbach’s alpha coefficient 0.839). Scores between 15 and 25 are considered to be within the normal range, and scores below 15 suggest low self-esteem [46].

#### Generalized anxiety disorder-7 (GAD-7)

Standardized rating scales such as the GAD-7are used to assess severity of symptoms of generalized anxiety disorder. The GAD-7 has also been used to diagnose and assess anxiety symptoms [48]. It comprises 7 items describing symptoms that participants may have experienced in the previous2 weeks (e.g., I feel nervous and upset). Responses to all items are on a 4-point scale (0 = totally none to 4 = almost every day). Total scores range from 0 to 28 (Cronbach’s alpha coefficient 0.922), with scores over 10 suggesting the presence of anxiety disorder [48].

#### Center for epidemiological studies depression scale (CESD)

Depressive symptoms were assessed with the CESD. Participants are asked 20 questions to assess the depressive symptoms they experienced in the previous 1 week (e.g., I feel sad.). Each item is scored from 0 to 3 (0 = rarely or none of the time, 1 = some or little of the time, 2 = moderately or much of the time, and 3 = most or almost all the time). Items 4, 8, 12, and 16 are reversed in valence. Scores for each item are summed to provide a total score. The scores ranged from 0 to 60 (Cronbach’s alpha coefficient 0.891). Higher scores indicate more severe depressive symptoms. Scores over 16 were used to define major depressive symptoms.

#### University of California, Los Angeles Loneliness Scale (UCLA Loneliness Scale)

Loneliness symptoms were assessed using the 8-item UCLA Loneliness Scale (e.g., “I feel isolated”). Responses are on a 4-point Likert scale ranging from 0 to 3. Items 3 and 6 were reversed in valence (Cronbach’s alpha coefficient 0.829). Scores over 18 suggest loneliness symptoms.

#### Sexual compulsivity scale

Sexual compulsivity was assessed with 10 questions or statements (e.g., “I think of sex when I am working”). Item 1 is reversed in valence (Cronbach’s alpha coefficient 0.860). Scores over 26 indicate sexual compulsivity symptoms.

#### Syndemic of psychosocial factors

If more than two psychosocial problems occurred at the same time in an individual participant, we assumed there was a syndemic phenomenon. A syndemic variable was created by counting the number of psychosocial factors.

#### Unprotected anal intercourse

Participants were asked questions about condom use when having anal sex with male sex partners in the past 6 months. We used a Likert scale to measure condom use, with response options ranging from 1 (never) to 5 (always). Men who responded with a number other than 5 (always) were considered to have participated in unprotected sex when having anal sex.

## Results

### Participants’ characteristics

In total, 547 participants completed the questionnaire. Table 1 presents the participants’ demographic characteristics. The age of participants ranged from 17.33 to 65.33 years (mean = 30.50 years; SD = 8.84 years). Those aged 25 to 40 years accounted for 61.6% of participants. The majority (71.3%) of participants went to college, indicating that most participants had a background of higher education; 79.3% were single and 15.0% were married to a woman. Around 24.3% of participants earned less than 3000 RMB per month (approximately 488 USD), and 73.1% came from outside Shanghai. Of the participants, 71.3% identified themselves as homosexual and 6.8% of participants self-reported being HIV positive. In addition, the prevalence of UAI in the past 6 months was 54.5%.

**Table 1:** Socio-demographic characteristics of men who have sex with men in Shanghai, China (*n* = 547)

Characteristics	*n*	%
Age (years)	(17.33–65.33)	
< 25	148	27.1
25–40	337	61.6
> 40	62	11.3
Education		
High school	157	28.7
College	390	71.3
Marital Status		
Single	434	79.3
Married	82	15.0
Divorced or Widowed	31	5.7
Monthly income (CNY^a^)		
< 3000	133	24.3
3000–6000	211	38.6
> 6000	203	37.1
Residential status		
Local inhabitant	147	26.9
Non-local inhabitant	400	73.1
Self-report Sexual orientation		
Not homosexual	157	28.7
Homosexual	390	71.3
HIV Positive		
Yes	24	4.4
No	523	95.6
Unprotected anal intercourse (UAI)		
No	249	45.5
Yes	298	54.5

^a^CNY 3000 equivalent to 480 USD; 6000 equivalent to 960 USD

### Psychosocial factors

Table 2 describes the prevalence of participants’ individual psychosocial factors. Around 10.6% of participants reported having low self-esteem, 12.2% reported experiencing anxiety, 52.1% reported feeling depressed, and 24.2% reported feeling lonely. The prevalence of sexual compulsivity was 20.3%. Approximately 35.5% reported having more than two psychosocial problems, which were considered syndemic of the five psychosocial factors.

**Table 2:** Psychosocial factors of MSM in Shanghai, China (*n* = 547)

Factors	*N*	%
Rosenberg Self-Esteem Scale		
High level (Score ≥15)	489	89.4
Low level (Score <15)	58	10.6
GAD Anxiety		
Low level (Score <10)	480	87.8
High level (Score ≥10)	67	12.2
CESD		
Low level (Score <16)	262	47.9
High level (Score ≥16)	285	52.1
UCLA Loneliness Scale		
Low level (Score ≤18)	414	75.8
High level (Score >18)	132	24.2
Sexual compulsivity		
Low level (Score ≤26)	435	79.7
High level (Score >26)	111	20.3
Syndemic of five factors		
No	353	64.5
Yes	194	35.5

### Socio-demographic characteristics and associations with UAI

We used a univariate logistic regression to analyze the association between socio-demographic characteristics and UAI (Table 3). Those with a high educational background (graduate from college) were less likely to participate in UAI, and those with a poor educational background were more likely to do so (OR 0.59, 95% CI: 0.38–0.94). Participants with a monthly income between 3000 and 6000 were more likely to engage in UAI when compared with those with a monthly income less than 3000 (OR 1.90, 95% CI: 1.20–3.01).

**Table 3:** Associations between socio-demographic characteristics and unprotected anal intercourse among MSM in Shanghai, China

Characteristics	*N*	%	ORu (95% CI)
Age (years)			
< 25	76	51.4	1
25–40	187	55.5	1.06 (0.69–1.63)
> 40	35	56.5	1.10 (0.54–2.21)
Education			
High school	99	63.1	1
College	199	51.0	0.59 (0.38–0.94)*
Marital Status			
Single	233	53.7	1
Married	48	58.5	0.86 (0.50–1.50)
Divorced or Widowed	17	54.8	0.76 (0.34–1.70)
Monthly income (CNY^a^)			
< 3000	64	48.1	1
3000–6000	132	62.6	1.90 (1.20–3.01)*
> 6000	102	50.2	1.32 (0.79–2.20)
Residential status			
Local inhabitant	80	54.4	1
Non-local inhabitant	218	54.5	0.91 (0.60–1.37)
Self-reported Sexual orientation			
Not homosexual	91	58.0	1
Homosexual	207	53.1	0.86 (0.58–1.28)

*ORu* odds ratio obtained from forward stepwise univariate logistic regression

^a^CNY 3000 equivalent to 480 USD; 6000 equivalent to 960 USD; *p < 0.05

### Associations between psychosocial factors and UAI

After controlling for demographic factors such as education and monthly income, we used a binary logistic regression model to analyze the relationships between psychosocial factors and UAI. Table 4 shows that after controlling for demographic factors. Self-esteem was significant in univariate logistic regression. We conducted a multivariate logistic regression to evaluate the multiple psychosocial problems (including self-esteem) with UAI among MSM. The result showed that self-esteem remained significant (*P* = 0.009). However, most *p* values of other four psychosocial factors were above 0.05, indicating that the association between UAI and single psychosocial factors was ambiguous.

**Table 4:** Associations between psychosocial factors and UAI among MSM, Shanghai, China (*n* = 547)

Factors	*n*	%	ORu (95% CI)	AOR (95% CI)	ORm (95% CI)	P
Rosenberg Self-esteem						
High level (Score ≥15)	257	52.6	1	1		
Low level (Score <15)	41	70.7	2.18 (1.20–3.94)*	1.94 (1.06–3.56)*	2.19 (1.21-3.95)**	0.009
GAD Anxiety						
Low level (Score <10)	257	53.5				
High level (Score ≥10)	41	61.2	1.37 (0.81–2.31)	1.42 (0.83–2.42)	-	**-**
CESD						
Low level (Score <16)	137	52.3	1	1		
High level (Score ≥16)	161	56.5	1.19 (0.85–1.66)	1.19 (0.84–1.67)	-	**-**
UCLA Loneliness						
Low level (Score ≤18)	218	52.7	1	1		
High level (Score >18)	79	59.8	1.34 (0.90–2.00)	1.25 (0.83–1.88)	-	**-**
Sexual compulsivity						
Low level (Score ≤26)	228	52.4	1	1		
High level (Score >26)	69	62.2	1.49 (0.97–2.29)	1.42 (0.92–2.19)	-	**-**

*AOR* adjusted OR, odds ratios adjusted for education level and monthly income, *ORm* odds ratio obtained from forward stepwise multivariate logistic regression

*p < 0.05, **p < 0.01

If two or more than two psychosocial factors (syndemic of five psychosocial factors) occurred at the same time for a single participant, they were more likely to report engaging in UAI in the past 6 months (OR 1.65, 95% CI: 1.09–2.50; *P* = 0.018). As self-esteem was significant in both univariate and multivariate regression model, we tried to exclude self-esteem from the model to avoid its effects, and use other four psychosocial factors to verify the overlapping and reinforcing effects on UAI (syndemic of four psychosocial factors). Results still showed positive associations between these four syndemic factors and UAI among MSM, Shanghai, China (OR 1.52, 95% CI: 1.06–2.20) (Table 5).

**Table 5:** Associations between syndemic factors and UAI among MSM, Shanghai, China (*n* = 547)

	Number (%)	MSM who have UAI	
		*N* (row%)	AOR (95% CI)
Have a syndemic of five psychosocial factors			
No (have no more than 1 psychosocial problem)	353 (64.5)	177 (50.1)	1
Yes (have 2 or more psychosocial problems)	194 (35.5)	121 (62.4)	1.65 (1.09–2.50)*
Have a syndemic of four psychosocial factors exclude Self-esteem			
No (have no more than 1 psychosocial problem)	373 (68.2)	191 (51.2)	1
Yes (have 2 or more psychosocial problems)	174 (31.8)	107 (61.5)	1.52 (1.06–2.20)*

*p < 0.05

## Discussion

We found that more than half of the participants (54.5%) reported having had UAI in the past 6 months. This indicates that a high number of MSM in Shanghai have engaged in high-risk sexual behaviors, particularly as the prevalence of HIV was 6.8%. Our finding suggests an urgent need to promote safe sex among MSM to prevent HIV spread to the general population. This was consistent with findings from other studies [6, 9].

We found that a background of higher education was a protective factor against UAI. It may be that those with a better education understand more about HIV prevention. The association between monthly income and UAI was more complicated. Our findings indicated that to a certain level, the prevalence of UAI increased as monthly income increased. However, this pattern of behavior changed when monthly income exceeded a certain amount (6000RMB).

Most research on MSM in China has focused on high-risk sexual behaviors. There was a paucity of literature exploring psychosocial factors and their relationship to high-risk sexual behaviors. Our findings indicated a high prevalence of psychosocial problems among MSM in Shanghai, China. More than half of the participants (52.1%) suffered from depression and 35.5% had been diagnosed with at least two symptoms of psychosocial problems. Of these five psychosocial factors, lower self-esteem was associated with UAI in both univariate and multivariate regression model, while other factors were insignificant. Although the occurrence of a single psychosocial factor was not necessarily associated with UAI, there was a greater chance that he might engage in UAI (OR 1.65, 95% CI: 1.09–2.50; *P* = 0.018) if a participant suffered from more than two psychosocial factors. Even if we excluded self-esteem from the model and employed another univariate logistic regression analysis, the syndemic effect of psychosocial problems on UAI was still significant. This supported our hypothesis that the overlapping and reinforcing effect (syndemic) of psychosocial problems might exist in MSM in Shanghai, China. In addition, the co-occurrence of psychosocial factors may fuel high-risk sexual behaviors and HIV infection in MSM in Shanghai, China.

Most intervention strategies targeting this population subgroup focus on behavioral change. These strategies have only had a moderate effect in reducing high-risk behaviors for HIV [1, 49]. As the additive effect of psychosocial problems may reduce the effects of HIV preventive strategies, a holistic framework should be considered for this population. We observed associations between syndemic psychosocial factors, high-risk sexual behaviors, and HIV infection among MSM in Shanghai, China. As 35.5% of participants had more than two psychosocial problems, and this syndemic may increase high-risk sexual behaviors and HIV infection, a tailored preventive strategy should be implemented that addresses the compounding and amplifying effect between psychosocial factors as well as behavioral interventions.

Our study had several limitations. First, we used a cross-sectional design, and this limited the exploration of influential factors and the inference of causal influences. Second, we used a snow-balling method to identify eligible participants rather than a random trial. All participants were recruited from MSM who attended the Shanghai Youth AIDS Health Promotion Centre to have an HIV test, and therefore excluded those who were unwilling to have an HIV test. Therefore, in a future study we should expand the sample size to cover those who did not want access to a HIV test. Finally, because high-risk sexual behaviors such as UAI are sensitive topics in mainland China and all data were self-reported, participants may not have been completely truthful. Biases in self-reported data were inevitable; however, reliability and validity studies indicated a good test-retest and fit of the results for the measures used.

Despite these limitations, our findings showed that most MSM in Shanghai, China engaged in high-risk sexual behaviors, and suffered from psychosocial problems to some extent. A number of MSM also simultaneously experienced more than two kinds of psychosocial problems, which may have an additive effect. Our results provided evidence to support a holistic approach in intervention strategies targeted to this group that focus on psychosocial factors as well as behavior change. Our goal was not only to promote safe sex, but also to reduce psychosocial problems.

## Conclusions

MSM represents the largest number of the population infected with HIV. This group is also affected by various psychosocial problems. Many studies have suggested strong links between psychosocial burdens, high-risk sexual behaviors, and HIV infection, referred as a syndemic, with a mutually reinforcing (additive or multiplicative) nature. However, to date, little is known about the psychosocial problems in the MSM population in China. Our cross-sectional study investigated the prevalence of psychosocial problems and tested the syndemic theory among MSM in Shanghai, China, and determined the direction and magnitude of the associations between psychosocial factors and high-risk behaviors among MSM. Our findings may provide evidence to inform tailored preventive strategies and public health policies targeting at this population subgroup.

## Abbreviations

CDC: Center for disease control and prevention
CESD: Center for epidemiological studies depression scale
GAD-7: Generalized anxiety disorder-7
HIV: Human immunodeficiency virus
MSM: Men who have sex with men
MSP: Multiple sexual partners
OR: Odds ratio
SD: Standard deviation
UAI: Unprotected anal intercourse
UCLA Loneliness Scale: University of California, Los Angeles Loneliness Scale

## References

[1] Safren SA, Reisner SL, Herrick A, Mimiaga MJ, Stall RD (2010). Mental health and HIV risk in men who have sex with men. J Acquir Immune Defic Syndr.

[2] Thomas B, Mimiaga MJ, Kumar S, Swaminathan S, Safren SA, Mayer KH (2011). HIV in Indian MSM: reasons for a concentrated epidemic & strategies for prevention. Indian J Med Res.

[3] Bai X, Luo S, Wang X, Yang J, Fan S, Yu M (2014). [Change of risky sexual behaviors among men who have sex with men before and after recent identification of HIV diagnosis]. Zhonghua Liu Xing Bing Xue Za Zhi.

[4] Li HM, Peng RR, Li J, Yin YP, Wang B, Cohen MS (2011). HIV incidence among men who have sex with men in China: a meta-analysis of published studies. PLoS One.

[5] Berry M, Wirtz AL, Janayeva A, Ragoza V, Terlikbayeva A, Amirov B (2012). Risk factors for HIV and unprotected anal intercourse among men who have sex with men (MSM) in Almaty, Kazakhstan. PloS one.

[6] Chow EP, Chen X, Zhao J, Zhuang X, Jing J, Zhang L (2015). Factors associated with self-reported unprotected anal intercourse among men who have sex with men in Changsha city of Hunan province, China. AIDS Care.

[7] Yang Z, Zhang S, Dong Z, Jin M, Han J (2014). Prevalence of unprotected anal intercourse in men who have sex with men recruited online versus offline: a meta-analysis. BMC Public Health.

[8] Zhou Y, Li D, Lu D, Ruan Y, Qi X, Gao G (2014). Prevalence of HIV and syphilis infection among men who have sex with men in China: a meta-analysis. Biomed Res Int.

[9] Wu J, Hu Y, Jia Y, Su Y, Cui H, Liu H (2014). Prevalence of unprotected anal intercourse among men who have sex with men in China: an updated meta-analysis. PLoS One.

[10] Chinese Center for Disease Control and Prevention Report. HIV/AIDS Epidemic in China. 2015.

[11] Jie W, Ciyong L, Xueqing D, Hui W, Lingyao H (2012). A syndemic of psychosocial problems places the MSM (men who have sex with men) population at greater risk of HIV infection. PLoS One.

[12] Yu F, Nehl EJ, Zheng T, He N, Berg CJ, Lemieux AF (2013). A syndemic including cigarette smoking and sexual risk behaviors among a sample of MSM in Shanghai, China. Drug Alcohol Depend.

[13] Wim VB, Christiana N, Marie L (2014). Syndemic and other risk factors for unprotected anal intercourse among an online sample of Belgian HIV negative men who have sex with men. AIDS Behav.

[14] Diaz RM, Ayala G, Bein E, Henne J, Marin BV (2001). The impact of homophobia, poverty, and racism on the mental health of gay and bisexual Latino men: findings from 3 US cities. Am J Public Health.

[15] Mimiaga MJ, O’Cleirigh C, Biello KB, Robertson AM, Safren SA, Coates TJ (2015). The effect of psychosocial syndemic production on 4-year HIV incidence and risk behavior in a large cohort of sexually active men who have sex with men. J Acquir Immune Defic Syndr.

[16] Guadamuz TE, McCarthy K, Wimonsate W, Thienkrua W, Varangrat A, Chaikummao S (2014). Psychosocial health conditions and HIV prevalence and incidence in a cohort of men who have sex with men in Bangkok, Thailand: evidence of a syndemic effect. AIDS Behav.

[17] Mimiaga MJ, Noonan E, Donnell D, Safren SA, Koenen KC, Gortmaker S (2009). Childhood sexual abuse is highly associated with HIV risk-taking behavior and infection among MSM in the EXPLORE Study. J Acquir Immune Defic Syndr.

[18] De Santis JP, Colin JM, Provencio Vasquez E, McCain GC (2008). The relationship of depressive symptoms, self-esteem, and sexual behaviors in a predominantly Hispanic sample of men who have sex with men. Am J Mens Health.

[19] Wilson PA, Stadler G, Boone MR, Bolger N (2014). Fluctuations in depression and well-being are associated with sexual risk episodes among HIV-positive men. Health Psychol.

[20] Preston DB, D’Augelli AR, Kassab CD, Cain RE, Schulze FW, Starks MT (2004). The influence of stigma on the sexual risk behavior of rural men who have sex with men. AIDS Educ Prev.

[21] Halkitis PN, Kupprat SA, Hampton MB, Perez-Figueroa R, Kingdon M, Eddy JA, et al. Evidence for a Syndemic in Aging HIV-positive Gay, Bisexual, and Other MSM: Implications for a Holistic Approach to Prevention and Healthcare. Nat Resour Model. 2012;36(2). doi:10.1111/napa.12009. 10.1111/napa.12009 PMC3859453 24347817

[22] Ye J, Shim R, Rust G (2012). Health care avoidance among people with serious psychological distress: analyses of 2007 Health Information National Trends Survey. J Health Care Poor Underserved.

[23] Zhou ZH, Li SM, Liu YJ, Jiang SL, Zhang XX, Li QC (2010). [Study on the relationship between behavioral factors, psychological status and HIV infection among men who have sex with men in Beijing.]. Zhonghua Liu Xing Bing Xue Za Zhi.

[24] Parsons JT, Grov C, Golub SA (2012). Sexual compulsivity, co-occurring psychosocial health problems, and HIV risk among gay and bisexual men: further evidence of a syndemic. Am J Public Health.

[25] Operario D, Nemoto T (2010). HIV in transgender communities: syndemic dynamics and a need for multicomponent interventions. J Acquir Immune Defic Syndr.

[26] Stall R, Mills TC, Williamson J, Hart T, Greenwood G, Paul J (2003). Association of co-occurring psychosocial health problems and increased vulnerability to HIV/AIDS among urban men who have sex with men. Am J Public Health.

[27] O’Leary D (2014). The syndemic of AIDS and STDS among MSM. Linacre Q.

[28] Singer M, Clair S (2003). Syndemics and public health: reconceptualizing disease in bio-social context. Med Anthropol Q.

[29] Center for Disease Control and prevention. Milstein B. Introduction to the Syndemics Prevention Network. Atlanta: Centers for Disease Control and Prevention; 2002. Available: https://www.cdc.gov/nchhstp/programintegration/Definitions.htm. Accessed 16 Dec 2016.

[30] Meyer JP, Springer SA, Altice FL (2011). Substance abuse, violence, and HIV in women: a literature review of the syndemic. J Women’s Health.

[31] Mustanski B, Garofalo R, Herrick A, Donenberg G (2007). Psychosocial health problems increase risk for HIV among urban young men who have sex with men: preliminary evidence of a syndemic in need of attention. Ann Behav Med.

[32] Santos GM, Do T, Beck J, Makofane K, Arreola S, Pyun T (2014). Syndemic conditions associated with increased HIV risk in a global sample of men who have sex with men. Sex Transm Infect.

[33] Deuba K, Ekstrom AM, Shrestha R, Ionita G, Bhatta L, Karki DK (2013). Psychosocial health problems associated with increased HIV risk behavior among men who have sex with men in Nepal: a cross-sectional survey. PLoS One.

[34] Lin H, Ding Y, Liu X, Wu Q, Shen W, He N (2015). High prevalence of HIV infection and bisexual networks among a sample of men who have sex with men in eastern china. PLoS One.

[35] Chen X, Li X, Zheng J, Zhao J, He J, Zhang G (2015). Club drugs and HIV/STD infection: an exploratory analysis among men who have sex with men in Changsha, China. PloS one.

[36] Chen H, Li Y, Wang L, Zhang B (2015). Causes of suicidal behaviors in men who have sex with men in China: a national questionnaire survey. BMC Public Health.

[37] Li D, Li C, Wang Z, Lau JT (2015). Prevalence and associated factors of unprotected anal intercourse with regular male sex partners among HIV negative men who have sex with men in China: a cross-sectional survey. PLoS One.

[38] Dyer TP, Shoptaw S, Guadamuz TE, Plankey M, Kao U, Ostrow D (2012). Application of syndemic theory to black men who have sex with men in the multicenter AIDS cohort study. J Urban Health.

[39] Mimiaga MJ, Biello KB, Robertson AM, Oldenburg CE, Rosenberger JG, O’Cleirigh C (2015). High prevalence of multiple syndemic conditions associated with sexual risk behavior and HIV infection among a large sample of Spanish- and Portuguese-speaking men who have sex with men in Latin America. Arch Sex Behav.

[40] le Giang M, Viet VD, Hao BT (2012). Sexual health and men who have sex with men in Vietnam: an integrated approach to preventive health care. Adv Prev Med.

[41] Herrick AL, Lim SH, Plankey MW, Chmiel JS, Guadamuz TE, Kao U (2013). Adversity and syndemic production among men participating in the multicenter AIDS cohort study: a life-course approach. Am J Public Health.

[42] Cantwell J, Muldoon O, Gallagher S (2015). The influence of self-esteem and social support on the relationship between stigma and depressive symptomology in parents caring for children with intellectual disabilities. J Intellect Disabil Res.

[43] Tucker A, Liht J, de Swardt G, Jobson G, Rebe K, McIntyre J (2014). Homophobic stigma, depression, self-efficacy and unprotected anal intercourse for peri-urban township men who have sex with men in Cape Town, South Africa: a cross-sectional association model. AIDS Care.

[44] Hubach RD, Dodge B, Li MJ, Schick V, Herbenick D, Ramos WD (2015). Loneliness, HIV-related stigma, and condom use among a predominantly rural sample of HIV-positive men who have sex with men (MSM). AIDS Educ Prev.

[45] Taylor SE, Brown JD (1988). Illusion and well-being: a social psychological perspective on mental health. Psychol Bull.

[46] Robins RW, Hendin HM, Trzesniewski KH (2001). Measuring Global Self-Esteem: Construct Validation of a Single-Item Measure and the Rosenberg Self-Esteem Scale[J]. Person Soc Psychol Bull.

[47] Rosenberg M (1965). Society and the adolescent self-image.

[48] Swinson R (2006). The GAD-7 scale was accurate for diagnosing generalized anxiety disorder. Evid Based Med.

[49] Ferlatte O, Hottes TS, Trussler T, Marchand R (2014). Evidence of a syndemic among young Canadian gay and bisexual men: uncovering the associations between anti-gay experiences, psychosocial issues, and HIV risk. AIDS Behav.

